# Comparison of early changes in tear film protein profiles after small incision lenticule extraction (SMILE) and femtosecond LASIK (FS-LASIK) surgery

**DOI:** 10.1186/s12014-024-09460-1

**Published:** 2024-02-17

**Authors:** Petri Mäkinen, Janika Nättinen, Ulla Aapola, Juhani Pietilä, Hannu Uusitalo

**Affiliations:** 1 SILK, Eye and Vision Research, Faculty of Medicine and Health Technology, Tampere University, Tampere, Finland; 2 Silmäasema Eye Hospital, Hämeenkatu 6, Tampere, 33100 Finland; 3 TAUH Eye Center, Tampere University Hospital, Tampere, Finland

**Keywords:** Refractive surgery, Tear, Proteomics, SMILE, Femtosecond laser, LASIK

## Abstract

**Background:**

Small incision lenticule extraction (SMILE) and femtosecond laser-assisted in situ keratomileusis (LASIK) are widely used surgical methods to correct myopia with comparable efficacy, predictability, and safety. We examined and compared the early changes of tear protein profiles after SMILE and FS-LASIK surgery in order to find possible differences in the initial corneal healing process.

**Methods:**

SMILE operations for 26 eyes were made with Visumax femtosecond laser. In FS-LASIK surgery for 30 eyes, the flaps were made with Ziemer FEMTO LDV Z6 femtosecond laser and stromal ablation with Wavelight EX500 excimer laser. Tear samples were collected preoperatively, and 1.5 h and 1 month postoperatively using glass microcapillary tubes. Tear protein identification and quantification were performed with sequential window acquisition of all theoretical fragment ion spectra mass spectrometry (SWATH-MS).

**Results:**

Immediately (1.5 h) after we found differences in 89 proteins after SMILE and in 123 after FS-LASIK operation compared to preoperative protein levels. Of these differentially expressed proteins, 48 proteins were common for both surgery types. There were, however, quantitative differences between SMILE and FS-LASIK. Upregulated proteins were mostly connected to inflammatory response and migration of the cells connected to immune system. One month after the operation protein expressions levels were returned to baseline levels with both surgical methods.

**Conclusions:**

Our study showed that immediate changes in protein profiles after SMILE and FS-LASIK surgeries and differences between the methods are connected to inflammatory process, and the protein levels quickly return to the baseline within 1 month. The differences in protein profiles between the methods are probably associated with the different size of the epithelial wound induced.

**Supplementary Information:**

The online version contains supplementary material available at 10.1186/s12014-024-09460-1.

## Background

Femtosecond laser in situ keratomileusis (FS-LASIK) and small incision lenticule extraction (SMILE) are widely used surgical methods to correct myopia and myopic astigmatism with comparable efficacy, predictability, and safety [1–3]. In FS-LASIK surgery, a stromal flap is made on the cornea with femtosecond laser and refractive correction is made with excimer laser reshaping of the underlying stroma [4, 5]. In SMILE surgery, an intrastromal lenticule is created with femtosecond laser and then removed through a small corneal incision [6].

In all keratorefractive techniques, surgery produces significant stress to the anterior structures of the eye. Corneal refractive surgery initiates an immediate healing response, which is a complex interplay between epithelial and stromal cells, corneal nerves, tear film and cells of the immune system [7–10]. The wound healing response after the operation is an important determinant of the outcomes and side effects. The response depends on the technique used and varies also according to patient-specific features, such as the amount of the treated refraction [11, 12].

Compared to FS-LASIK, the superficial cornea is left almost intact after SMILE surgery, as it induces less epithelial trauma with smaller incision size and lacking manipulation of the flap [13]. This approach results in SMILE having less effect on corneal biomechanics [14, 15] and corneal nerves [16–18]. Because of this, SMILE surgery may cause less postoperative neurotrophic issues, such as dry eye, compared to flap-based LASIK surgery [19, 20].

The tear fluid is an important mediator in wound healing process after corneal surgery and it can be collected non-invasively and analyzed with different methods after refractive surgery [21, 22]. We have previously studied early protein changes in tear fluid after uneventful FS-LASIK surgery using sequential window acquisition of all theoretical mass spectra method (SWATH-MS), which offers a tool to identify and quantify hundreds of proteins from small tear samples [23, 24]. The study showed that protein profile changes during the immediate postoperative recovery phase were connected to increased cell migration of immune cells and inflammatory response [24].

In the present study, we investigated the changes of tear fluid proteomics in the early stages after SMILE and FS-LASIK surgery. Tear fluid samples were collected with microcapillary tubes, and we used SWATH-MS method to quantify the tear proteins. Our aim was to find possible differences in the early healing processes after FS-LASIK and SMILE surgeries, which may eventually help us to understand the biological processes initiated by the surgical methods, individual variations of them and find more definite targets for initial phase treatment after surgery.

## Patients and methods

### Patients

Patients undergoing SMILE or FS-LASIK surgery were asked to take part in the study. After preoperative examination and discussion with the operating surgeon the surgery type was selected according to patient preference. The patients were not randomized to the treatment groups. In total 56 patients were included in the study and of those, 26 were treated with SMILE surgery and 30 with FS-LASIK surgery.

Written consent was obtained from all patients. The study was approved by the Ethical Committee of Pirkanmaa Hospital District (R13074) and followed the guidelines of the Declaration of Helsinki.

### Preoperative examination

All patients underwent a complete ophthalmic examination, which included biomicroscopy, evaluation of refraction, measurements of uncorrected and corrected distance visual acuity (UDVA and CDVA, respectively), measurement of corneal thickness and three-dimensional corneal topography (Allegro Oculyzer, Wavelight AG, Erlangen, Germany), wavefront analysis (Allegro Analyzer, Wavelight AG, Erlangen, Germany) and measurement of intraocular pressure (Nidek Tonoref RKT-7700, Gamagori, Aichi, Japan). Patients discontinued wearing contact lenses a minimum of one week before the surgery.

### Surgical technique

In both SMILE and FS-LASIK groups the pre- and postoperative medication protocols were identical. Prior to the surgery the following eye drops were instilled: to constrict conjunctival vessels, brimonidine tartrate 2 mg/ml (Alphagan, Allergan, Westport, Ireland); for pain and inflammation, diclofenac sodium 1 mg/ml (Voltaren Ophtha, THEA, Clermont-Ferrand, France); antibiotics, levofloxacin 5 mg/ml (Oftaquix, Santen Oy, Tampere, Finland); topical anaesthetic, oxibuprocain hydrochloride 4 mg/ml (Oftan Obucain, Santen Oy). The eyelid was opened with an aspirating speculum (Geuder, no 15,961, Heidelberg, Germany).

In the SMILE surgery, the Visumax femtosecond laser (Carl Zeiss Meditec, Jena, Germany) was used to create an intrastromal refractive lenticule and peripheral corneal incision. The Visumax had a repetition rate of 500 kHz and a pulse energy of 130 nJ. The cap thickness was 120 to 130 μm, and the cap diameter 7.9 mm. The lenticule diameter varied from 6.5 to 7.0 mm. A peripheral corneal incision varied from 2.8 to 3.0 mm. A thin blunt spatula was used to go through the incision and dissect the intrastromal lenticule. Special SMILE forceps were used to remove the lenticule. Intrastromal pocket was flushed with balanced salt solution.

In FS-LASIK surgery, the FEMTO LDV Z6 I femtosecond laser (Ziemer Ophthalmic Systems, Port, Switzerland), was used for the flap creation. FEMTO LDV had a repetition rate of > 2 MHz and 100 nJ pulse energy. The target flap thickness ranged from 90 to 110 μm and all flaps were roundly shaped and with a 60–90º angled edge. Plastic single-use suction rings with a 9.5 mm diameter were used with a target flap diameter of 9.3 mm. The target hinge length was 4.0 mm. After the flap lift, the excimer laser treatment was done on the exposed stroma using the Wavelight EX500 excimer laser (WaveLight AG, Erlangen, Germany). The optical zone ranged from 6.5 to 7.0 mm.

### Postoperative treatment and examination

Moisture drops sodium hyaluronate 0.15 mg/ml (Oxyal, Dr Gerhard Mann Chem. -pharm. Fabrik GmbH, Berlin, Germany) were instilled onto the eyes 30 min after the surgery. Topical anaesthetic drops oxibuprocain hydrochloride (Oftan Obucain, Santen Oy) were instilled one hour after the operation. Chloramphenicol and dexamethasone containing drops (Oftan Dexa-Chlora, Santen Oy) were started 3 h after the operation and were used for one week with tapered dose. Moisturizing eye drops were used as needed for the following month. The frequency of the moisturizing drops was not monitored in the study. First postoperative examination was done 1,5 h after the operation before the discharge from the clinic and the second approximately 1 month after the operation. Perioperative complications were registered in both of these postoperative examinations. At 1 month visit, the examinations were the same as conducted preoperatively, excluding the wavefront analysis.

### Tear fluid sample processing and proteomic analysis

**Fig. 1:**
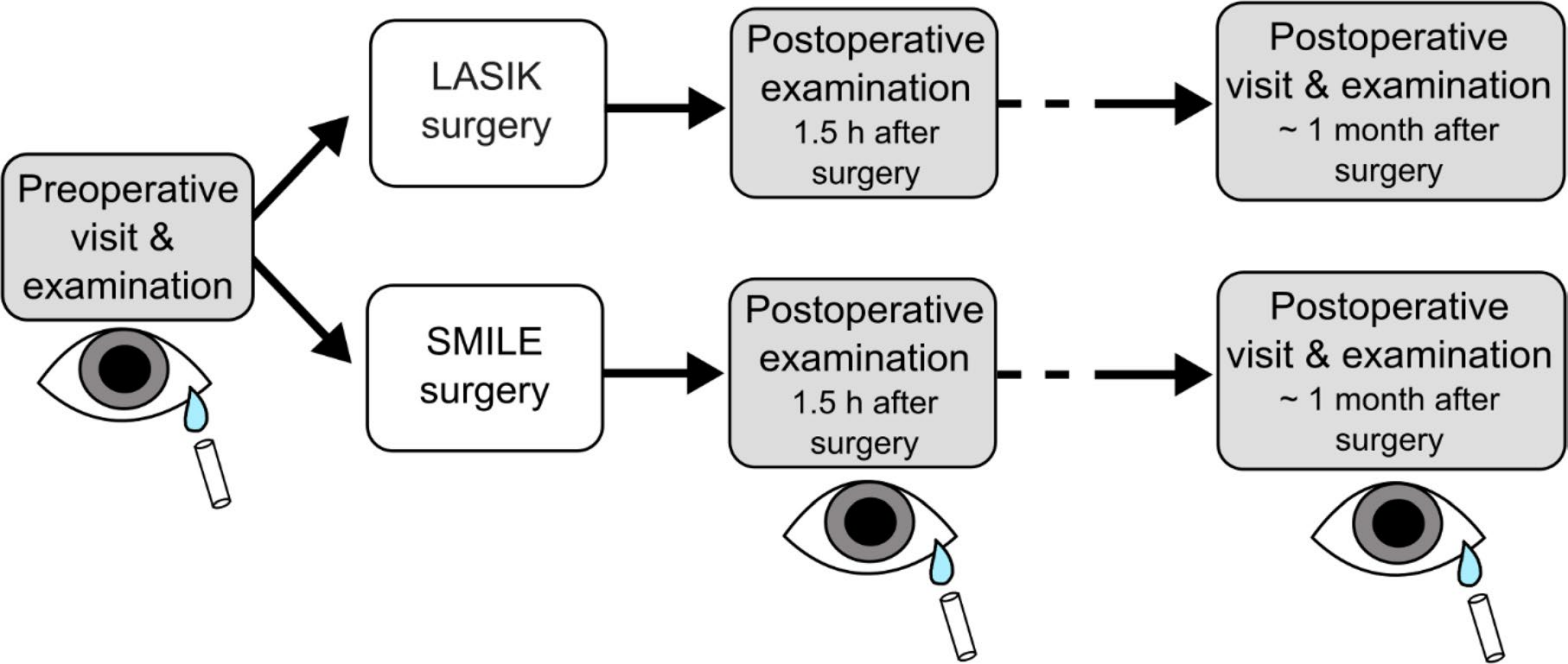
Outline of the study structure

Tear samples were collected into 2 or 3 µl glass microcapillary tubes from the inferior tear meniscus, avoiding contact with lower lid. The samples were collected before the surgery and postoperatively 1.5 h and 1 month after the operation (Fig. 1). The preoperative and 1 month tear samples were taken before any eye drops were installed. The samples were stored at -80 ºC until the analysis. Tear sample preparation is described in our previous publications [24, 25]. Briefly, tear fluid was flushed out from capillary tube and total protein concentration was measured with Bio-Rad DC protein quantification kit (Bio-Rad, Hercules, CA, USA). After acetone-precipitation, reduction, and alkylation on 30 kDa molecular weight cut-off filters (Pall Corporation, Port Washington, NY, USA) tear proteins were trypsin-digested overnight. Finally, digested peptides were cleaned and desalted using C18 tips (Thermo Fisher Scientific, Waltham, MA, USA) and equal amount (3 µg) of each sample was analyzed with Nano-LC-TripleTOF 5600 + mass spectrometer (Sciex, Concord, Canada) using SWATH acquisition method. Two replicate MS analyses were performed for each sample. In-house tear fluid peptide spectral library was utilized to identify the proteins in samples and quantification was performed with PeakViewer and MarkerViewer softwares (Sciex, Redwood City, CA, USA). Detailed protocols for SWATH-MS analysis, tear protein identification and quantification have been described in our previous publications [24, 25].

### Statistical analysis

The summary statistics for preoperative and postoperative clinical characteristics were displayed as mean ± standard deviation for continuous variables and as counts for nominal variables. Statistical significance was evaluated using Wilcoxon rank sum test for continuous and Fisher’s exact test for nominal variables. The nonparametric test was deemed more appropriate for continuous variables after careful inspection of normality using the Shapiro-Wilk normality test.

In proteomics data, 1028 proteins were quantified from 56 surgery patients (samples from 2 to 3 time points and 2 replicate MS runs each). Log_2_-transformation and central median normalization were applied to the data and protein expression level means of replicate MS runs were calculated for each sample and these values were used in further analyses.

For the preoperative tear proteomics, Wilcoxon rank sum test was used to evaluate the differences between FS-LASIK and SMILE surgery patients. Due to significant differences in preoperative tear protein levels between the surgery types, further analyses were conducted separately for SMILE and FS-LASIK groups. More specifically, the tear protein changes between preoperative and postoperative time points were conducted using Wilcoxon signed rank test. In addition to separate analyses for SMILE and FS-LASIK, analysis of covariance (ANCOVA) was also performed to study the specific differences between the study groups, while also accounting for the group-wise differences in baseline protein levels. Pathway analyses were carried out using the statistically significant proteins, i.e., those with fold change > 1.5 or < 0.67 and adjusted p-value < 0.05. Pathway analyses excluded disease-specific terms, such as ‘cancer’ and ‘tumor’.

Benjamini-Hochberg procedure was used to correct for multiple testing. The significance level was set at 0.05 unless otherwise stated. All statistical analyses for the proteomics data were performed using R software version 4.1.2 (R Foundation for Statistical Computing, Vienna, Austria). IPA (Ingenuity Pathway Analysis) software (IPA, QIAGEN Redwood City) was used in the consecutive pathway analyses.

## Results

The demography of the patients and characteristics of the preoperative ocular measurements are presented in Table 1. There were no statistical differences between the study groups.

**Table 1:** Preoperative clinical information of the FS-LASIK and SMILE patients

Clinical variable	FS-LASIK (*n* = 30)	SMILE (*n* = 26)	P-value
Age (years)	29.9 ± 9.9	30.8 ± 8.3	0.39
Sex (male/female)	17/13	14/12	0.89
SEQ (D)	-3.6 ± 2.1	-3.8 ± 1.4	0.33
Sphere (D)	-3.2 ± 2.3	-3.5 ± 1.5	0.39
Cyl (D)	-0.8 ± 0.8	-0.6 ± 0.6	0.57
K1 (D)	43.5 ± 1.2	43.9 ± 1.4	0.40
K2 (D)	44.4 ± 1.2	44.9 ± 1.4	0.15
IOP	15.4 ± 3.4	15.6 ± 4.0	0.75
CDVA	1.0 ± 0.1	1.0 ± 0.1	0.47
Preop pachymetry (µm)	533.0 ± 30.7	549.3 ± 28.6	0.07

The values are shown as mean ± standard deviation for continuous variables and as counts for nominal variables. Statistical significance was evaluated using Wilcoxon rank sum test for continuous and Fisher’s exact test for nominal variables. D = diopter; SEQ = spherical equivalent refraction; Cyl = cylinder error, astigmatism; K1 and K2 = corneal keratometry; IOP = intraocular pressure; CDVA = corrected distance visual acuity

The clinical results 1 month after the surgery are summarized in Table 2. Both techniques were comparable in predictability of achieving target spherical equivalent refraction, but the mean postoperative astigmatism was larger after SMILE vs. FS-LASIK, respectively (-0.13 ± 0.25 vs. -0.03 ± 0.14, *p* = 0.008). RST was higher after FS-LASIK vs. SMILE (375 ± 32.1 vs. 340 ± 35.4, *p* = 0.0009).

**Table 2:** Clinical results 1 month after the surgery

Clinical variable	FS-LASIK (*n* = 30)	SMILE (*n* = 26)	p-value
SEQ (D)	0.15 ± 0.38	-0.01 ± 0.52	*
Sphere (D)	0.16 ± 0.36	0.08 ± 0.52	*
Deviation from target SEQ (D)	0.2 ± 0.3	0.2 ± 0.3	0.65
Cyl (D)	-0.03 ± 0.14	-0.13 ± 0.25	**0.008**
K1 (D)	40.5 ± 2.0	40.7 ± 1.9	0.66
K2 (D)	41.5 ± 2.0	41.6 ± 1.8	0.62
IOP	11.3 ± 2.3	11.0 ± 2.3	0.35
CDVA	1.1 ± 0.1	1.0 ± 0.1	0.07
RST (µm)	375.1 ± 32.1	340 ± 35.4	**0.0009**
Postop pachymetry (µm)	466.4 ± 34.5	476.7 ± 34.3	0.35

The values are shown as mean ± standard deviation. D = diopter; SEQ = spherical equivalent refraction; Cyl = cylinder error, astigmatism; K1 and K2 = corneal keratometry; RST = residual stromal thickness; IOP = intraocular pressure; CDVA = corrected distance visual acuity

* Because mild myopic target of SEQ and Sphere in some eyes, statistical analysis was not done

Bold value: The significance level was set at 0.05

### Preoperative differences in tear proteomics between SMILE and FS-LASIK patients

**Fig. 2:**
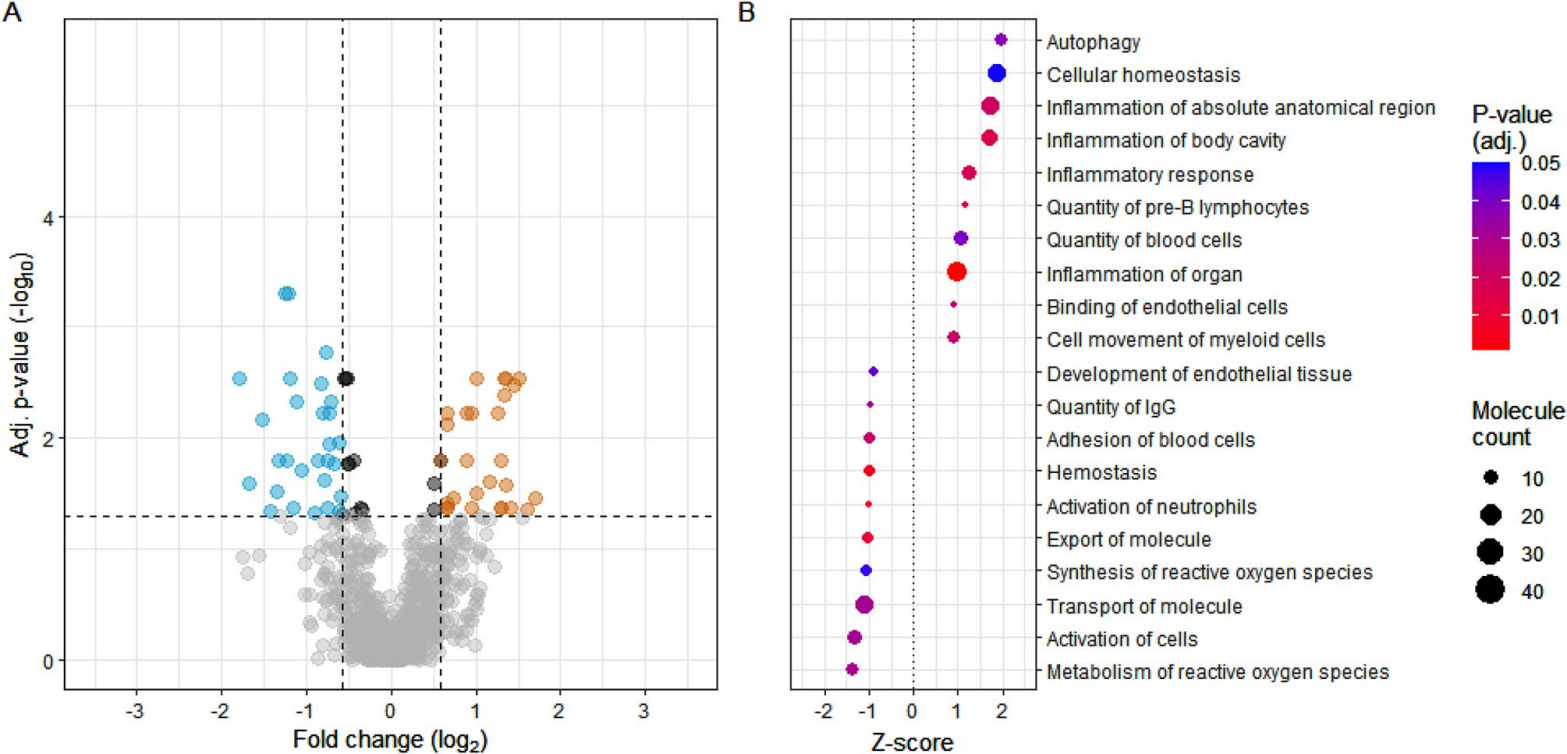
The differences between SMILE and FS-LASIK groups’ preoperative tear protein levels. Volcano plot visualizes the statistically significant proteins based on log_2_ fold change (x-axis) and adjusted p-value (y-axis) (**A**). The proteins that were less- or more abundant in SMILE patients in comparison to FS-LASIK patients are colored blue and orange, respectively. The top 10 significantly active and inhibited biological functions (in SMILE patients) associated with the statistically significant proteins are further visualized in the dotplot (**B**)

Fifty-five proteins differed significantly between the surgery types prior to the surgery, i.e., had adjusted p-value < 0.05 and fold change > 1.5 or < 0.67. Of these proteins, 28 were down- and 27 upregulated and the results are further visualized in Fig. 2A. The differences in preoperative tear levels also indicated that individuals undergoing SMILE surgery had higher levels of autophagy and inflammation but also cellular homeostasis and lower levels of reactive oxygen species related functions, when compared to individuals undergoing FS-LASIK (Fig. 2B).

### Effects of SMILE and FS-LASIK surgeries on tear proteomics

**Fig. 3:**
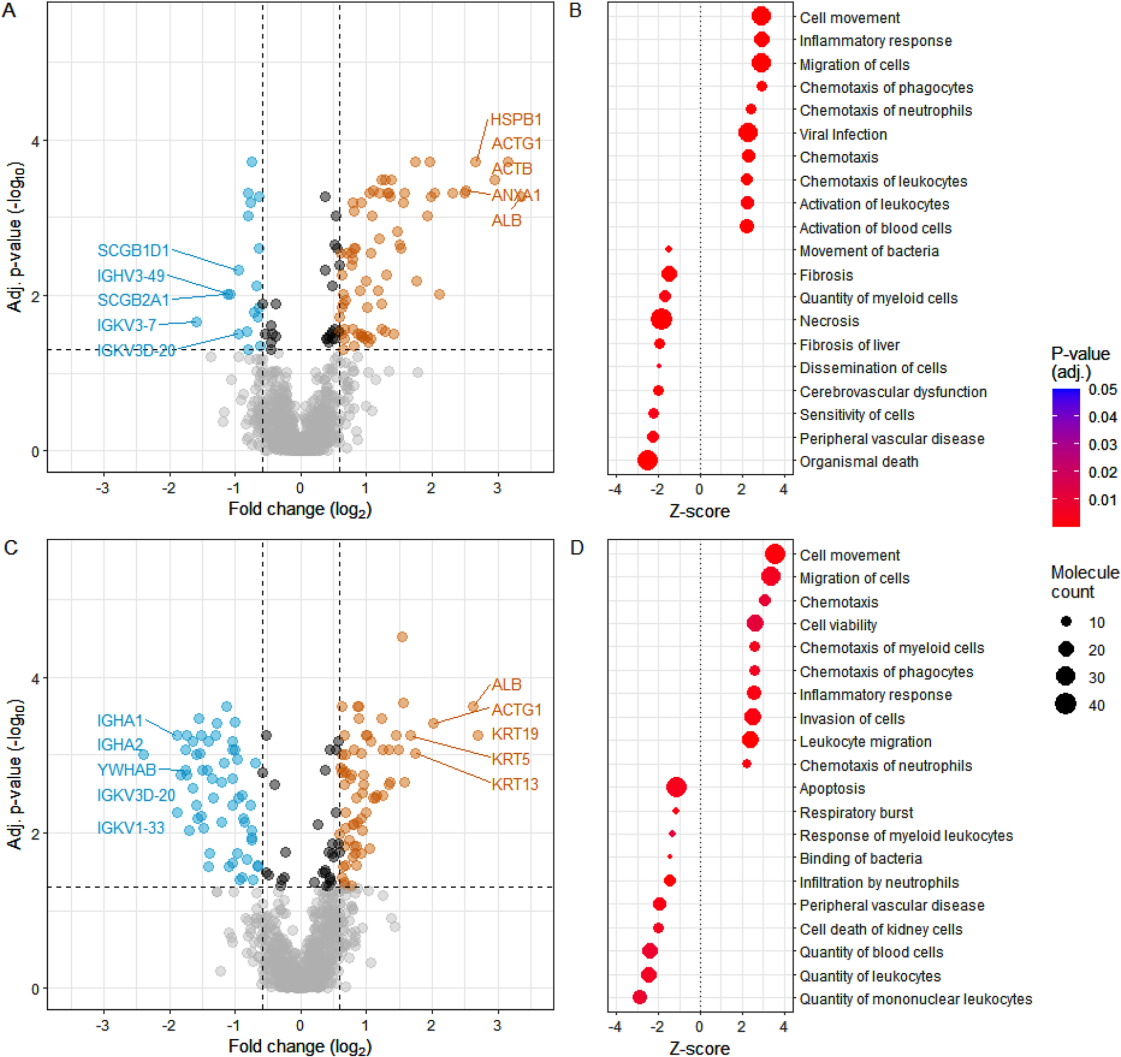
Differences between preoperative and 1.5-hours postoperative tear protein levels for SMILE (**A**, **B**) and FS-LASIK (**C**, **D**). Volcano plots visualize the statistically significant proteins based on log_2_ fold change (x-axis) and adjusted p-value (y-axis) when preoperative and immediate postoperative tear levels are compared for SMILE (**A**) and FS-LASIK (**C**). The proteins down- and upregulated 1.5 h after the surgery in comparison to the preoperative time point are colored blue and orange, respectively. The five most up- and downregulated proteins are further labeled for both surgery types. The top 10 activated and inhibited biological functions associated with the statistically significant proteins are visualized by dotplots for SMILE (**B**) and FS-LASIK (**D**)

Due to the preoperative differences in the tear protein levels between the groups, we could not compare post-surgery groups directly with each other but inspected the changes in protein levels separately in SMILE and FS-LASIK groups. Consequently, the numbers of differentially expressed proteins are not directly proportional to each other. Immediate postoperative tear protein levels, i.e., 1.5 h after the surgery, differed significantly from the preoperative tear levels for both SMILE and FS-LASIK patients. More specifically, 1.5 h after SMILE operations 89 proteins and after FS-LASIK operations 123 proteins differed from the preoperative protein levels. Altogether 48 of these differentially expressed proteins were common for both surgery types. However, were found some quantitative differences in protein expression levels between the techniques (Additional file 1). Visualizations of the results with top 5 named upregulated and downregulated proteins of each technique are displayed in Fig. 3A and C. Of the top 5 upregulated proteins of each technique, keratins (KRT) 5, 13 and 19 upregulated after FS-LASIK surgery, were found equally upregulated after SMILE. Albumin (ALB), Heat shock protein beta-1 (HSPB1), Annexin A1 (ANXA1) and cytoplasmic actins (ACTB and ACTG1) were upregulated in both techniques, but upregulation was more pronounced after SMILE. Of the top downregulated proteins, several immunoglobulins and 14-3-3 protein beta/alpha (YWHAB) were more downregulated after FS-LASIK.

The differentially expressed proteins were further analyzed with IPA to evaluate what biological functions are activated and inhibited based on the tear protein level changes. For SMILE, activation, migration and chemotaxis of immune cells as well as inflammatory response were activated 1.5 h after the surgery, while organismal death, necrosis and fibrosis were inhibited (Fig. 3B). For FS-LASIK, similar functions, i.e., immune cell movement and inflammatory response, were activated while apoptosis and quantities of immune cells were inhibited (Fig. 3D). Overall, no large differences between the surgery types were observed immediately after the surgery according to tear protein levels.

**Fig. 4:**
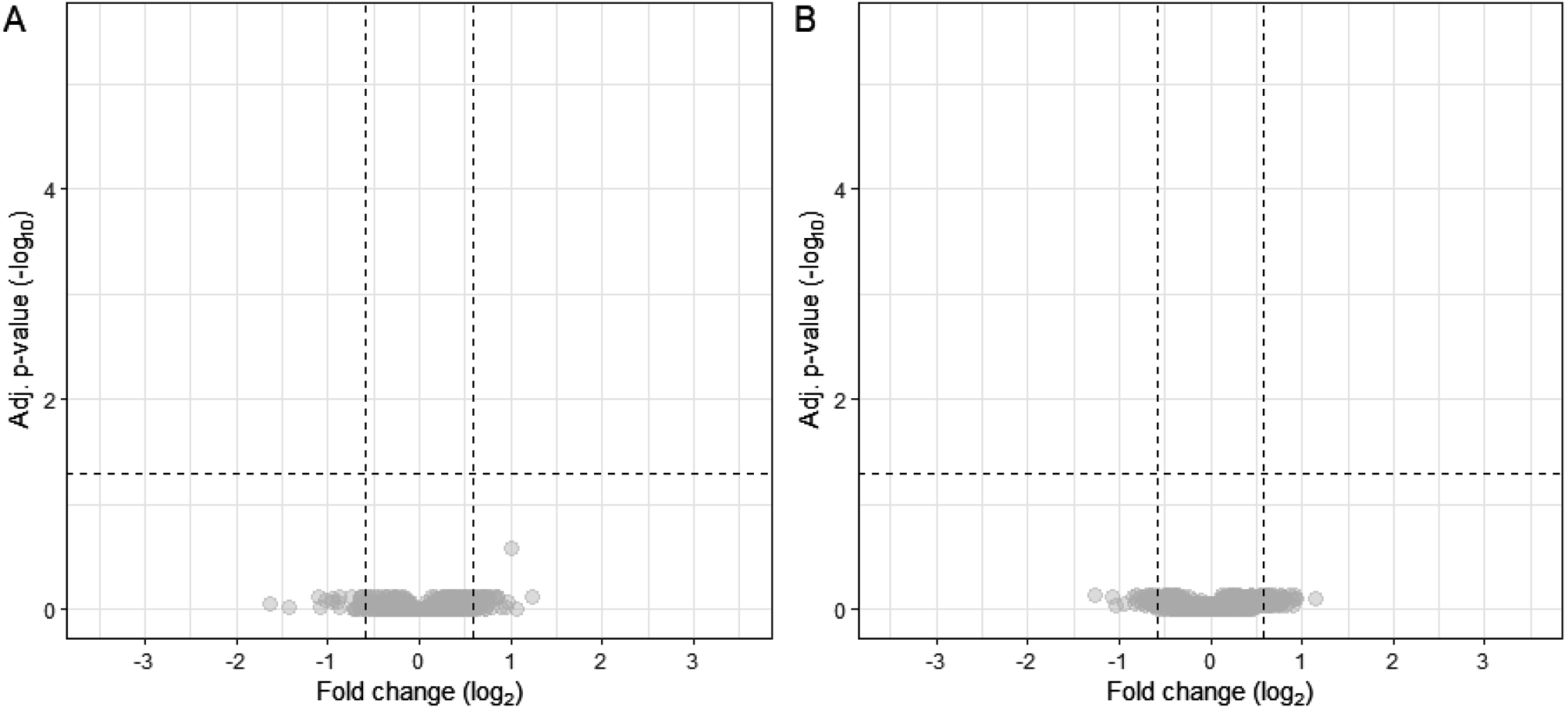
Differences between preoperative and 1-month postoperative tear protein levels for SMILE (**A**) and FS-LASIK (**B**). Volcano plots visualize no statistically significant proteins based on log_2_ fold change (x-axis) and adjusted p-value (y-axis) when preoperative and 1-month postoperative tear levels are compared for SMILE (**A**) and FS-LASIK (**B**)

When we compared the tear protein levels between the preoperative and 1-month postoperative visits, no significant proteins were found, i.e. the differential protein expressions levels returned to baseline in both surgery type groups (Fig. 4).

### ANCOVA analyses

In order to overcome the effect of baseline differences on the tear film proteomic results we performed ANCOVA analyses for 1.5 h and 1-month timepoints. With ANCOVA it is possible to statistically take into account the effect of the baseline differences between the groups. This method revealed few proteins which had statistical differences at these postoperative timepoints (Table 3).

**Table 3:** ANCOVA analysis of differences in protein levels comparing SMILE vs. FS-LASIK treated eyes at different time points postoperatively

Postoperative time point	Protein name	Gene name	Fold change (log_2_)	Adj. p-value
1,5 h	Keratin, type I cytoskeletal 9	KRT9	-1.63	**0.043**
	Protein OS9	OS9	-0.84	**0.030**
	Insulin-like growth factor-binding protein complex acid labile subunit	IGFALS	0.85	**0.043**
1 month	Alpha-1-antitrypsin	SERPINA1	-0.75	**0.047**
	Procollagen-lysine,2-oxoglutarate 5-dioxygenase 1	PLOD1	-0.97	**0.045**
	Complement C1q subcomponent subunit C	C1QC	1.43	**0.045**
	Cytosolic Fe-S cluster assembly factor NUBP2	NUBP2	1.22	**0.045**

The fold change is negative, when protein level is higher in FS-LASIK group and positive, when protein level is higher in SMILE group

## Discussion

In the present study, we compared the early changes in tear fluid proteomics collected with microcapillaries after two keratorefractive surgery types, FS-LASIK and SMILE. We demonstrated an immediate response of proteins related to cell movement, inflammatory process, cell migration, activation and chemotaxis of inflammatory cells. This immediate response in protein expression levels returned to baseline within 1 month in both surgery types.

### Clinical findings

Clinical results 1 month after the surgery were comparable between the techniques, although some differences were detected. Postoperative astigmatism was found higher in SMILE group than in the FS-LASIK group. Similar difference in accuracy of astigmatism correction have been reported earlier [2, 26]. The recovery of corneal optical quality is known to be slightly slower after SMILE [27], and some difference was found in CDVA compared to FS-LASIK. Residual corneal thickness of the stromal bed was less in SMILE group due to technical difference between the procedures. The surgery is made deeper into the corneal stroma, and in addition the same amount of refractive correction removes thicker part of corneal stroma in SMILE surgery compared to FS-LASIK surgery.

### Proteomic findings

Immediately (1.5 h) after the surgery, 89 proteins in SMILE group and 123 proteins in FS-LASIK group were up- or downregulated in comparison to the preoperative levels. Although the individual proteins behaved generally similarly after the operation, some differences in the magnitude of the expression level changes were found. Among the upregulated proteins, ALB, HSPB1, ANXA1, ACTB and ACTG1 were more upregulated after SMILE. Of these ALB, ANXA1 and ACTB have previously been seen upregulated in aqueous-deficient or Sjögren’s syndrome related dry eyes [28, 29]. ANXA1 is an anti-inflammatory protein secreted by corneal and conjunctival epithelial cells which regulates homeostasis of the ocular surface. It stimulates mucin secretion from conjunctival goblet cells, contributing to the good mucin layer of the tear film [30–32]. In femtosecond laser based refractive surgeries there are also differences between the devices in the suction systems which positions the eyeball during the laser cut [33]. The Visumax femtosecond laser used in SMILE treatments generates suction on the cornea near the limbus. In contrast, FEMTO LDV which was used in our study in FS-LASIK treatments, applies a suction on the limbal conjunctiva and sclera. Therefore, the compression caused by the suction was distributed differently on the corneal and conjunctival epithelial cells and goblet cells. This may result in differences in the postoperative protein expressions. HSPB1 responds to environmental stress and has an important role in corneal epithelial protection and wound healing [34, 35]. The role of ACTG1 in postoperative healing period is not clear, but it has previously been associated with keratoconus and treatment effect of dry eye [36, 37]. These findings may partly indicate the preoperative difference between groups observed in this study but partly be related to differences in the surgical techniques.

Some proteins were also decreased 1,5 h after the surgery. In FS-LASIK group this protein group consisted mainly immunoglobulin subunits. Immunoglobulin downregulation was also found in SMILE group, but in lesser extent. Immunoglobulins are important local agents of a defense mechanism in tear fluid, which are earlier found to be rather increased in different ocular diseases [38]. Immediate decrease of immunoglobulins after FS-LASIK was detected also in our earlier study [24]. We hypothesized that in the initial stage of the wound healing the immunoglobulins may be bound to the surgically wounded tissue and therefore they are less detected in the tear fluid. Larger LASIK-flap and thus larger epithelial defect was also found to be correlated to greater downregulation of immunoglobulins 1,5 h after the operation [24]. The surgically induced epithelial defect after SMILE is smaller and that may result in less tissue bounded immunoglobulins and more immunoglobulins in tear fluid compared to FS-LASIK.

The differences in tear fluid proteomics between the two methods were minimal and could be at least partly explained by the baseline differences. With ANCOVA analyses we tried to overcome that disparity. Only few proteins had statistical differences and two of them awoke clinical interest. Of those proteins, KRT9 was more upregulated in FS-LASIK group 1.5 h after the operation. KRT9 is typically highly specific keratin of terminally differentiating keratinocytes of palmoplantar epidermis [39], but has previously been found upregulated at high levels in pterygium and pinguecula [40]. In our study, the patients did not have pterygiums or pingueculae preoperatively, but the more intense suction and irritation on the perilimbal area during the flap creation in FS-LASIK may explain the difference between the groups. SERPINA1 was more upregulated in FS-LASIK group with ANCOVA analysis 1 month after the operation. The level of SERPINA1 in tear fluid have been reported to be increased in patients with corneal ulcers, conjunctival diseases and after continuous contact lens wear [41, 42]. That may indicate more active healing process in FS-LASIK treated eyes still after 1 month when compared to SMILE operated eyes.

When the changes in protein profiles were analyzed with IPA, the biological functions did not differ significantly between SMILE and FS-LASIK. Biological processes connected to wound healing, such as inflammatory response and activation, movement and chemotaxis of immune cells, were found to be activated after both techniques 1.5 h postoperatively. In corneal wound healing, keratocyte apoptosis is detected in minutes after epithelial injury, and it is found to be most prominent at 4 h after injury [10]. It is the main form of cell death after LASIK surgery and appears to continue for at least 1 week [43]. Unexpectedly, in our study IPA analysis showed that in FS-LASIK group 1.5 h after the surgery tear protein levels connected to apoptosis were downregulated. Although apoptosis is the first wound healing response, which is followed by other processes, many of these events occur simultaneously. In our study, the decrease in apoptosis connected proteins in tear fluid was also detected in SMILE group, but not significantly. We may speculate that in tear fluid level the intensive immediate healing response, particularly apoptosis, is exhausted 1.5 h after the surgery. This difference between groups could be explained by more pronounced healing response and exhaustion after FS-LASIK surgery due to larger epithelial defect and additional excimer laser ablation compared to SMILE. The site of the apoptosis may also explain the decreased markers in tears in both techniques. Keratocyte apoptosis after LASIK surgery is detected most typically in the corneal stroma, anterior and posterior to the lamellar interface [43, 44].

SWATH-MS provides accurate and reproducible quantification data for a large number of proteins in a single experiment, making it valuable tool in clinical research concentrating on comparative proteomics [21]. To our knowledge there is only one previous study, which compared larger scale tear fluid proteomics following SMILE and FS-LASIK surgery [45]. Liu and co-workers examined protein profiles preoperatively and 1 week, 1, 3, 6 and 12 months postoperatively by using a similar type of SWATH-MS method as in our study. Contrary to our results they found a persistent changes in their tear samples as late as 12 months after the procedures. One explanation to this difference could be the fact that they used a different sampling method, Schirmer strips instead of microcapillaries. Previous studies have demonstrated the impact of the tear sampling method on the tear proteomics [25, 46, 47]. When protein profiles obtained with SWATH-MS were compared in previous studies, microcapillary samples produced higher expression levels of extracellular proteins and Schirmer strips yielded higher expression levels of cell- and organelle-originating proteins [25]. This is most probably due to the fact, that Schirmer’s strips are in addition to tear fluid collecting also epithelial cells of palpebral conjunctiva into the samples. The difference in our and Liu’s studies may indicate that the processes initiated after keratorefractive surgery are staying active longer in the epithelial tissues than in tear fluid. We selected microcapillary tubes as a collection method, because 1,5 h after the surgery eyes are sensitive for mechanical irritation and there is a risk for epithelial defect or even flap displacement caused by the mechanical irritation of Schirmer strip. Most probably because of this, in the study of Liu et al., the first samples were collected later, 1 week after the operations [45]. Thus, the collection method and timing have a crucial role when the tear fluid proteomics after refractive surgery are studied and compared.

In refractive surgery, topical medication is an essential part of the operation and perioperatively used eyedrops potentially have at least short-term effect on the biological functions of the ocular surface as well as the tear fluid composition and protein profiles. The topical eyedrops are important but unavoidable factors when the changes in protein profiles are evaluated [24]. However, the pre- and postoperative medication was identical with both surgical techniques, so there should not be eye drop produced bias on protein alterations between FS-LASIK and SMILE.

One of the limitations in our study was that the patients were not randomized to treatment groups. In meta-analyses SMILE is associated to have lower risk of postoperative dry eye symptoms than FS-LASIK [48, 49]. In one review article SMILE surgery is suggested to be preferred choice in patients with mild dry eye disease [50]. That may conduct patient and surgeon when the surgical technique is chosen. The patients who ask to be treated with SMILE surgery have commonly more corneal surface related symptoms than FS-LASIK attracted patients. Pre- and postoperative tear break-up time and Schirmer’s test were not measured in our study. The grading of possible dry eye would have helped to analyze the differences between the groups. However, the demographics of the groups, e.g., age and preoperative refraction were comparable (Table 1). That is important, because the tear physiology, such as composition, secretion, volume and stability, is known to change with age [51]. Similarly, we have shown earlier that several tear fluid proteins connected to inflammation are affected by increasing age [52]. The amount of myopic correction in FS-LASIK surgery also affects several proteins closely connected to inflammation [24].

In the present study, the preoperative difference in patient groups reflected in preoperative tear protein levels. Autophagy and inflammation related biological functions were upregulated in SMILE group when compared to FS-LASIK group. Dysregulated autophagy has been implicated to different corneal diseases and inflammation, e.g., dry eye [53]. As mentioned earlier, this baseline difference may also explain some of the postoperative difference in protein changes between the groups. Overall, our study suggests that despite of the preoperative condition of the eye and surgical method used, the major transformation in tear protein profiles occur immediately after the operation.

## Conclusions

Our study showed that there is an immediate change in tear fluid protein profiles and analyzed biological functions after FS-LASIK and SMILE surgery. However, after both methods, the recovery to the preoperative levels is fast and almost complete during the first postoperative month. Some variation was found in proteins connected to inflammatory process, probably indicating the difference in surgically induced epithelial defect between the methods.

As the healing process continues longer than 1 month and clinical symptoms take up to one year or at least several months to recover from refractive surgery, the tear fluid proteomics with microcapillary tear samples and SWATH-MS method may not reveal all the proteins connected to the healing cascade. In addition to tear proteomics, we encourage utilizing also other methodology to study the recovery process.

### Electronic supplementary material

Below is the link to the electronic supplementary material.

Additional file 1 : Title and description of data: Wilcoxon signed rank test (paired) was performed for SMILE and LASIK groups separately between preoperative and 1.5 hours postoperative timepoints. Statistically significant proteins (adj. P-value < 0.05) for either group are listed.

## Data Availability

The datasets used and analyzed during the current study are available from the corresponding author on reasonable request.

## Abbreviations

ACTB: actin, cytoplasmic 1
ACTG1: actin, cytoplasmic 2
ALB: albumin
ANCOVA: analysis of covariance
ANXA1: annexin A1
CDVA: corrected distance visual acuity
FS-LASIK: Femtosecond laser in situ keratomileusis
HSPB1: Heat shock protein beta-1
IPA: Ingenuity Pathway Analysis
KRT: keratin
KRT9: keratin, type I cytoskeletal 9
LASIK: laser in situ keratomileusis
MS: mass spectrometry
SERPINA1: alpha-1-antitrypsin
SMILE: Small incision lenticule extraction
SWATH: sequential window acquisition of all theoretical fragment ion spectra
SWATH-MS: sequential window acquisition of all theoretical fragment ion spectra mass spectrometry
UDVA: uncorrected distance visual acuity
YWHAB: 14-3-3 protein beta/alpha
